# Changes in Socioeconomic Inequalities in Amenable Mortality after the Economic Crisis in Cities of the Spanish Mediterranean Coast

**DOI:** 10.3390/ijerph17186489

**Published:** 2020-09-06

**Authors:** Pamela Pereyra-Zamora, José M. Copete, Adriana Oliva-Arocas, Pablo Caballero, Joaquín Moncho, Carlos Vergara-Hernández, Andreu Nolasco

**Affiliations:** 1 Research Unit for the Analysis of Mortality and Health Statistics, Department of Community Nursing, Preventive Medicine, Public Health and History of Science, University of Alicante, 03080 Alicante, Spain; copetealacant@yahoo.co.uk (J.M.C.); adriana.oliva@ua.es (A.O.-A.); pablo.caballero@ua.es (P.C.); joaquin.moncho@ua.es (J.M.); nolasco@ua.es (A.N.); 2 Área de Desigualdades en Salud, Fundación para el Fomento de la Investigación Sanitaria y Biomédica de la Comunitat Valenciana (FISABIO), 46035 Valencia, Spain; vergara_car@gva.es

**Keywords:** mortality, amenable mortality, socioeconomic factors, economic recession, small-area analysis, Spain

## Abstract

Several studies have described a decreasing trend in amenable mortality, as well as the existence of socioeconomic inequalities that affect it. However, their evolution, particularly in small urban areas, has largely been overlooked. The aim of this study is to analyse the socioeconomic inequalities in amenable mortality in three cities of the Valencian Community, namely, Alicante, Castellon, and Valencia, as well as their evolution before and after the start of the economic crisis (2000–2007 and 2008–2015). The units of analysis have been the census tracts and a deprivation index has been calculated to classify them according to their level of socioeconomic deprivation. Deaths and population were also grouped by sex, age group, period, and five levels of deprivation. The specific rates by sex, age group, deprivation level, and period were calculated for the total number of deaths due to all causes and amenable mortality and Poisson regression models were adjusted in order to estimate the relative risk. This study confirms that the inequalities between areas of greater and lesser deprivation in both all-cause mortality and amenable mortality persisted along the two study periods in the three cities. It also shows that these inequalities appear with greater risk of death in the areas of greatest deprivation, although not uniformly. In general, the risks of death from all causes and amenable mortality have decreased significantly from one period to the other, although not in all the groups studied. The evolution of death risks from before the onset of the crisis to the period after presented, overall, a general pro-cyclical trend. However, there are population subgroups for which the trend was counter-cyclical. The use of the deprivation index has made it possible to identify specific geographical areas with vulnerable populations in all three cities and, at the same time, to identify the change in the level of deprivation (ascending or descending) of the geographical areas throughout the two periods. It is precisely these areas where more attention is needed in order to reduce inequalities.

## 1. Introduction

Amenable mortality (AM), understood as untimely and unjustified deaths that should not occur in the presence of timely healthcare procedures to avoid them, is a type of mortality used to assess the impact of the response and quality of a health system as well as the potential weaknesses of its healthcare. Thus, it has also been used during the last decades to evaluate the positive impact on a population’s health due to the improvements in access, monitoring, diagnosis, and treatment, particularly in industrial countries [1,2].

For decades, in most European countries the trend of all-cause mortality has been decreasing [3]. Moreover, a progressive decrease in amenable mortality can also be observed in several of these countries at different rates, depending on the country and population group [4,5,6]. However, in some of the lower-income European countries, this trend has tended to change direction in recent years, particularly in the case of women [7].

In this context, the impact of the economic downturn on health, either due to worsening general socioeconomic conditions, or due to cutbacks in health services and public investment in health, or the privatization of health services, is the subject of a growing scientific literature, whose results are paradoxical. On the one hand, a series of studies indicate that mortality has a pro-cyclical behaviour against macroeconomic difficulties; that is, the recession, unemployment, etc., cause an improvement in certain healthy habits; such as quitting smoking, cooking at home, playing sports, or visiting family and friends that improve living conditions and reduce mortality, while economic booms increase mortality [8,9]. On the other hand, economic crises can exacerbate poverty levels or stress and therefore increase morbidity and mortality in a counter-cyclical trend. Sometimes pro-cyclical and counter-cyclical effects operate sequentially [10] or at different rhythms, in the short and long term [7]. Some authors who provide pro-cyclical results warn that while a recession can reduce death rates in the general population, they can worsen in specific social sectors or geographical areas [11]. This shows the need to study socioeconomic inequalities in health in general, and in mortality in particular [12,13].

Within this growing scientific interest, various studies have investigated the impact of the economic slowdown on the population’s health and healthcare, both in Spain [14,15,16] and in other European countries [7,17,18,19], as well as in other continents [20,21]. The 2008 economic crisis coincided with the implementation of austerity policies that reduced the capacity of the Spanish public health system. This reduction struck unevenly depending on the position of the people and social groups in the social structure and depending on geographical location (rural/urban, centre/peripheral, outskirts, etc.). Therefore, as amenable mortality depends directly on the response capacity of the health system, its use is not only relevant as an indicator of the crisis impact, but also as an indicator of the inequalities of that impact at different socioeconomic or educational levels, sex/gender, age, ethnic group, or geographical area; so reveal recent studies in Spain [6,22] and Europe [23].

These inequalities in socioeconomic level or access to health services are in themselves a risk factor, and therefore it is necessary to study them in order to identify the most vulnerable groups or geographical areas to carry out specific interventions [24]. An adequate instrument to study health inequalities and the effects of economic downturns is the deprivation index (DI). Designed to measure the disadvantages of an individual, a family, or a group with regard to their community, or society, they are usually built from various indicators [25]. In Spain, a DI has been devised within the framework of the MEDEA projects [26]. This index, based on census data, has allowed the census tracts to be classified according to their level of socioeconomic deprivation, and its usefulness has been demonstrated in several studies on inequalities in mortality in urban areas [19,27,28].

In Europe, some studies on socioeconomic inequalities in amenable mortality at the country level or comparisons between countries have been carried out [4,29,30]. However, few studies have researched these inequalities at the urban level, and there is no evidence that the changes in these inequalities have been studied after the start of the 2008 economic slowdown. Therefore, the objective of this article is to analyse the socioeconomic inequalities in amenable mortality in the three most important cities of the Valencian Community (Spain), and their evolution after the start of the 2008 economic crisis, taking the census tract as the basic geographic unit.

The main hypothesis is that the economic crisis did not affect all social groups in the same way. This differentiation in impact might depend on multiple factors, ranging from the duration of the crisis in the different economic areas to the position of the different census tracts in the socioeconomic structure, and that of the families and individuals that inhabit them; also, the different actors’ responses (State, institutions, political parties, unions, families, and individuals) vis-a-vis the crisis and the crisis victims’ needs.

## 2. Materials and Methods

### 2.1. Design, Study Population, and Unit of Analysis

This is an ecological analysis of AM comparing two periods: 2000–2007 and 2008–2015. The units of analysis were the census tracts (CTs) of the cities of Alicante (178 CTs), Castellon (58 CTs), and Valencia (531 CTs). A census tract, in the different countries where it is used, is the smallest territorial unit, established for operational purposes, for which statistical data is available. In Spain, a CT average population is 1000 inhabitants. These three cities are located in the Autonomous Community of Valencia, with an average annual total population (in all three cities) of 1,240,744 inhabitants during the period 2000–2007 and 1,310,123 in the period 2008–2015.

### 2.2. Mortality Data

All deaths of residents in these cities in the study periods have been included in the research. The death data were taken from the Mortality Registry of the Valencian Community, obtaining the variables year of death, age, sex, city (Alicante, Castellón, and Valencia), and cause of death. The causes of death used in the analysis were coded according to the International Classification of Diseases, Tenth Revision (ICD-10). The causes of amenable deaths analysed in the study were those proposed by Nolte and McKee [1] (see Table A1 of Appendix A), and following the criteria defined by these authors. It is important to notice that only 50% of the deaths due to ischaemic heart disease were included [2,31]. All deceases were georeferenced and assigned to their CT of residence. The data were obtained from an anonymized database maintained by the Mortality Registry of the Autonomous Community of Valencia. Since the study was based on retrospective administrative data, the approval of an ethics committee in Spain was not required.

### 2.3. CTs by Socioeconomic Deprivation Level

A deprivation index (DI) for each CT, in all three cities and periods, was established using the following indicators (in percentage): (i) unemployment, (ii) manual workers, (iii) casual workers, (iv) insufficient education in young people (16 to 29 years), and (v) insufficient education in general. These indicators have already been proposed in the calculation of deprivation index (DI) on the basis of census data in major Spanish cities as the first component of a principal component analysis [26]. For our research, indicator data were obtained from the 2001 Population and Housing Census for the period 2000–2007, and from the 2011 Population and Housing Census for the period 2008–2015. The deprivation index used was developed within the framework of the MEDEA3 project (third edition of the national coordinated MEDEA project) from which the study data, both on socioeconomic inequality and mortality, stem.

For each period and city, the 10 (P10), 25 (P25), 75 (P75), and 90 (P90) DI percentiles were calculated. Thus, classifying the census tracts into five deprivation levels (DL) according to their value; that is, DL1, DI values lower than P10; DL2, DI values between P10 and P25; DL3, DI values between P25 and P75; DL4, DI values between P75 and P90; and DL5, DI values greater than P90.

**Figure 1:**
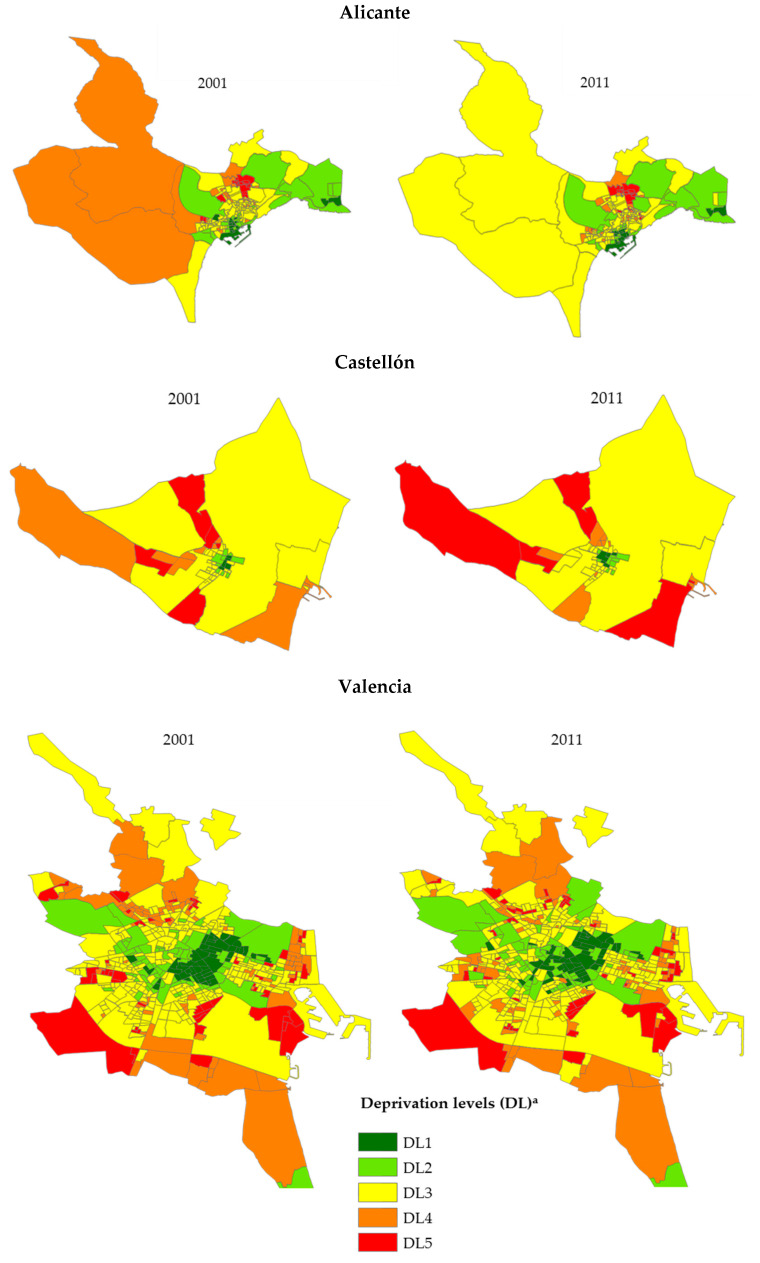
Geographical distribution of the five levels of deprivation (DL)^a^ according to census tracts in the cities of Alicante, Castellón, and Valencia (2001 and 2011).

Figure 1 shows the census tract distribution in the three cities in relation to their DL. This classification was outlined according to the aim of this research in order to quantify the difference in risks between the most socioeconomically favoured areas (DL1) and those of greatest deprivation (DL5). Table A2 of Appendix A shows the average values of the five socioeconomic indicators used in the different DLs of each city and period under study. In addition, the DI calculated for the two periods has made visible the changes that have occurred over time in the three cities (see Figure 1).

### 2.4. Population Data

The population data (by CT, year, age, and sex) used in order to calculate mortality indicators (rates and the relative risks) for the periods studied were obtained with permission from the Valencian Institute of Statistics, which is responsible for compiling population statistics in this region. Table A3 of Appendix A shows the average annual population for all the cities under study by sex, age group, DL, and period.

### 2.5. Data Analysis

To study the evolution of the risk of death over time, the data were classified into two periods: 2000–2007 (P1) and 2008–2015 (P2). Deaths were also grouped by three age ranges: 0–44, 45–64, and 65 and older.

The specific rates by sex, age group, DL, and period have been calculated for the total number of deaths due to all causes and the total amenable mortality. In order to estimate the relative risks (RRs) between the categories of the variables under study, the Poisson regression models also have been adjusted, taking into consideration the city, age, DL, and period effects, separated by sex, and carrying out a robust estimation to control the possible over-dispersion of the data. In addition, the proportional mortality of the large ICD-10 groups was calculated according to sex and deprivation level for all three cities so as to compare the pattern of mortality by groups of causes according to period. Finally, the program IBM^®^ SPSS^®^ Statistics (v.25) (Armonk, NY, USA) and our own software were used for calculating the mortality indicators.

## 3. Results

Between 2000 and 2015 there occurred 177,583 deaths in all three cities under study (40,774 in Alicante, 20,935 in Castellón, and 115,874 in Valencia). Nevertheless, 2634 of these (1.5%) could not be georeferenced and assigned to the census section of residence as the deceased person’s residence address was not stated or did not correspond to the cities under study. Regarding the remaining 174,949 that could be georeferenced, 86,479 occurred in the period 2000–2007 and 88,470 in 2008–2015. Table A4 and Table A5 of Appendix A show the death frequencies and percentages for the specific causes of amenable mortality and the chapters of the ICD-10, according to period, DL, and sex.

In Table 1, the average values and confidence interval of the DI are displayed. In it, it can be seen that the average values per DI varied scarcely from the period 2000–2007 to the period 2008–2015. The city of Castellón, for instance, showed smaller differences in the averages observed between the more extreme DLs, but similar in the rest of DLs. The table also includes the number of sections for each of the DLs in each city and all cities as a whole. Observing Table A2 of Appendix A, it can be noticed that areas with DL5 are areas with an alarming situation, where all the indicators used to build the index appear in high values: areas hit by unemployment, lack of training, school dropout, precarious work, and so on.

In order to verify if the effects of DL, period, and age group on mortality risk were significantly different according to city, the Poisson models were adjusted, including the effects of the following variables: city, DL, period, age group, and the interactions between the city and the rest of the other variables, verifying the absence of statistical significance of the terms of the interaction of the city effect with the other effects.

All interactions were not significant for both all-cause mortality (in men, *p* = 0.569 interaction with DL, *p* = 0.195 with period and *p* = 0.160 with age; in women *p* = 0.491 with DL, *p* = 0.070 with period and *p* = 0.101 with age) and mortality due to amenable causes (in men, *p* = 0.711 interaction with DL, *p* = 0.186 with period and *p* = 0.599 with age; in women *p* = 0.771 with DL, *p* = 0.632 with period and *p* = 0.072 with age). Due to the absence of a significant interaction, the estimation of effects was carried out jointly for the three cities under study.

**Figure 2:**
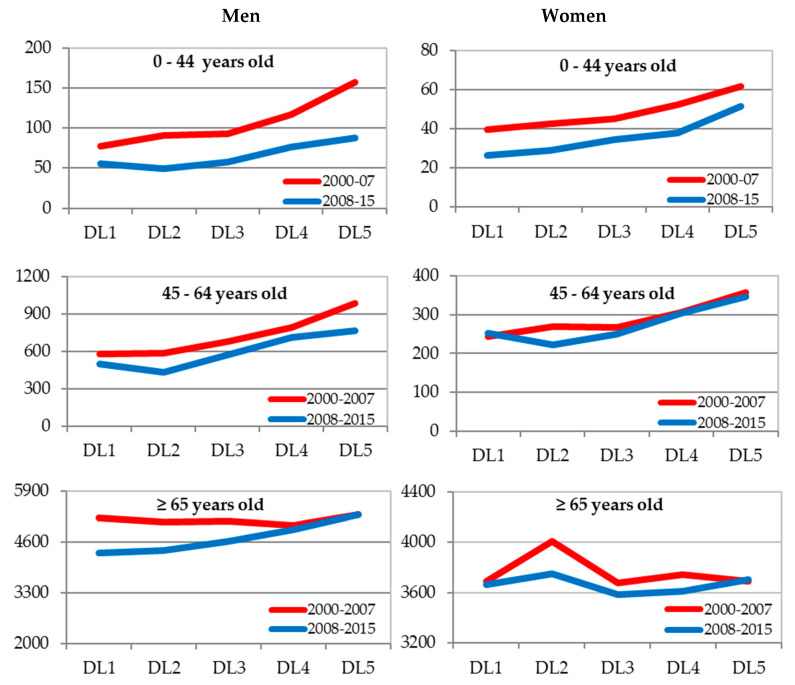
Specific mortality rates for all causes (×100,000) by sex, age, and deprivation level (DL). Alicante, Castellón, and Valencia jointly 2009–2015. DL: Deprivation level for the census tract of residence based on the deprivation index (DI). DL1: DI < P_10_; DL2: P_10_ ≤ DI < P_25_; DL3: P_25_ ≤ DI < P_75_; DL4: P_75_ ≤ DI < P_90_; DL5: DI ≥ P_90_. P_q_ = Percentile q.

**Figure 3:**
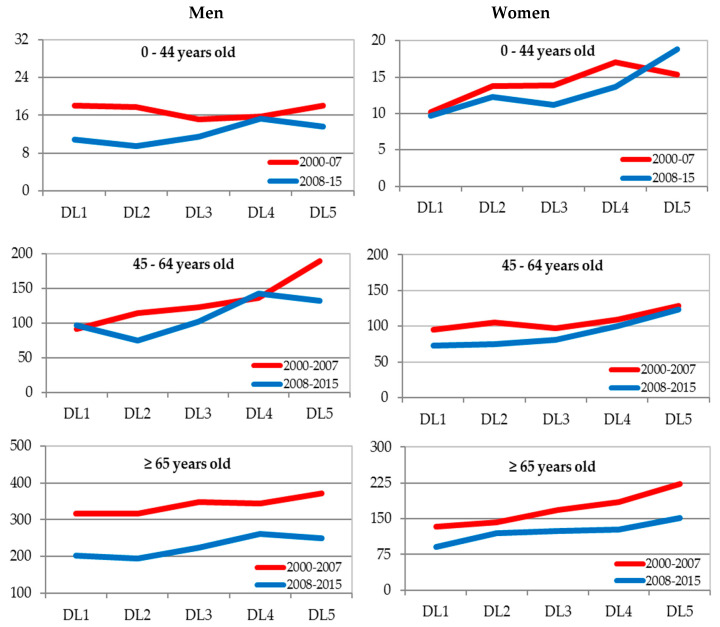
Specific mortality rates for amenable causes (×100,000) by sex, age, and deprivation level (DL) ^a^. Alicante, Castellón, and Valencia jointly 2009–2015. DL: Deprivation level for the census tract of residence based on the deprivation index (DI). DL1: DI < P10; DL2: P10 ≤ DI < P25; DL3: P25 ≤ DI < P75; DL4: P75 ≤ DI < P90; DL5: DI ≥ P90. P_q_ = Percentile q.

In the joint analysis of the three cities, the Poisson regression models were adjusted by sex. These included the effects of the following variables: DL, period, age group, the first-level interactions between DL and the rest of the other variables, and also the second-level interaction between DL, period, and age. These models suggested the existence of a significant (*p* < 0.05) second-level interaction between the DL effect, period, and age group in both men and women. Figure 2 and Figure 3 show the specific rates by sex, age group, period, and DL for all causes and amenable mortality (the values of the rates can be observed in Table A6 and Table A7 of Appendix A).

Mortality graphs for overall and amenable mortality suggest that the mortality rates are generally higher at the levels of greatest economic deprivation. The detected interaction could be due to some exceptions to this general behaviour. Thus, for general mortality in men in the age group of over 65 there are hardly any differences in rates according to the DL in the period 2000–2007, while, on the contrary, regarding amenable mortality in men aged 0–44 years, there are. In women, the age group 65 and over has not experienced increases in rates according to the DL for general mortality in any period, unlike for amenable mortality.

Due to the existence of an interaction, the relative risks between categories of DL (a measure of inequality according to DL) specific by sex, age, and period were estimated using a simple Poisson model with DL as the only effect. To estimate the increase or decrease in the risks of death of one to another period, a simple Poisson model specific by sex, age, and DL was adjusted with period as the only effect.

Regarding mortality from all causes, as Table 2 shows, the risk of death increased as the DL worsened, in the younger age groups (0–44 and 45–64 years), both in men and women (the significant RRs were greater than 1 in the highest categories, DL5 and DL4, when compared with DL1), and both in the first and second period under study. Nevertheless, in the 0–44 age group, the RRs were higher for men in the first period and women in the second, suggesting a tendency towards decreasing inequalities in men and increasing in women. The behaviour of the mortality risks in the age group of 65 years of age and over was different, since only the RRs significantly higher than 1 occurred in men in the second period, whereas regarding women only the relative risk of the DL2 group was significantly higher in the first period. Regarding the evolution from the first to the second period, overall, the risk of death decreased, with the RRs adjusted by age in the second period as compared to the first period of 0.875 (95% CI: 0.833–0.919) in men and 0.961 (95% CI: 0.945–0.977) in women.

Table 3 shows the RR of the 2008–2015 period vis-a-vis the 2000–2007 period. In men, a significant overall decrease in the risk of death in all categories of DL (except in DL4 and DL5 for the age group of 65 and over) can be seen. However, there was no significant drop in the risk of death at levels DL4 (ages 45–64 and 65 and more) and DL5 (all ages) and in DL1 (ages 45–64 and 65 and more) in women and in DL5 and DL4 (age 65 and over) in men. This means that men and women of these age groups and DL did not improve the risk of death from all causes.

Regarding mortality due to amenable causes, according to Table 4, the risks of death increased in women, for any age, in both periods, as the DL worsened. However, in men, the behaviour of this variable was different depending on the age group. In the group of 0–44 years of age, the RRs went from being lower than 1 (therefore lower risk of death in any category of DL than in DL1) in the first period to RRs greater than 1 in the worst DL categories (DL5 and DL4) in the second period. Although this suggests a tendency to increase inequality, these results were not significant. In addition, in the intermediate age group (45–64 years), the RRs were significantly higher than 1 in the most deprived DL categories (DL5 and DL4) in both periods. Finally, in the group of seniors (65 and over), the RRs increased slightly in the second period.

Comparing period 2008–2015 with period 2000–2007, it can be seen that the risk of death decreased, with RRs adjusted by age of 0.725 (CI95%: 0.659–0.798) in men and 0.785 (CI95%: 0.741–0.831) in women. Table 5 shows the RRs of the period 2008–2015 as compared to the period 2000–2007. A significant reduction in the risks of death in most of the DL categories can be observed, although with some exceptions, since no significant drop was observed in men of 0–44 years of age in DL1, DL4, and DL5 and of 45–65 years in DL1 and DL4, nor in women of 0–44 years in DL1, DL2, and DL4 and of 45–65 years in DL1, DL4, and DL5. In addition, there was an upsurge (not significant) in the risk of death (RR > 1) at the DL4 level in men of 45–64 years and in DL5 in women of 0–44 years.

## 4. Discussion

### 4.1. Summary of Findings: Inequalities and Evolution of Death Risk

This study has shown that the inequalities between areas of greater and lesser deprivation in both all-cause mortality and amenable mortality persist along the two study periods in the three cities, and that these inequalities appear with greater risk of death in the areas of greatest deprivation, although they present nuances depending on whether it is all-cause or amenable mortality, level of deprivation, age group, sex, or period. It has been found that, in general, the risks of death from all causes and amenable mortality have decreased significantly from one period to the other, although not in all the groups studied.

### 4.2. Inequalities

#### 4.2.1. Overall Mortality

Inequalities in all-cause mortality among levels of deprivation have not disappeared. In some cases, although inequalities remain, the RRs have decreased for both men and women, showing in most cases a clear gradient between the most impoverished and the most favoured levels. However, in some age groups, such as men 65 and over and women 0–44, inequalities have increased. In the case of younger men (0–44 and 45–64), inequalities tend to decrease. This result could indicate that men of working age are the recipients of pro-cyclical impacts on health. The reasons may be related to the reduction of work stress due to increased unemployment [32,33], in the specific Valencian case, due to the bursting of the housing bubble or a decrease in tobacco consumption [34], as well as the general decrease in pollution from industrial activity [35]. In other words, with the economic contraction, an overall reduction in mortality risks can be observed in men of working age. This process might have developed, to a greater extent, among the most deprived sectors, highly affected by unemployment. The analysis on the effects of pro-cyclical and counter-cyclical mechanisms proposed by Catalano et al. [33] is appropriate here.

In the case of men over 65, inequalities appear in the second period, while in the first period they were non-existent. This could be due to the fact that the economic crisis deteriorated the socioeconomic conditions of the census sections that already had high levels in all the deprivation indicators, in all three cities. This could have directly affected the age cohorts who had not yet retired, men in the later years of the working age—a situation aggravated by the feeling of not being able to fulfil the traditional provider role. This sector of men was most affected by the crisis, with deficiencies in unemployment benefits and in which the effects of this appear in the short but also in the long term, or even with permanent consequences of increased mortality, as found by Bender et al. in Greece [36].

In the case of women, inequalities persist, although not in all age groups. In women aged 65 and over, there are no inequalities in general mortality in either of the two periods, whereas in the youngest (0–44) these inequalities increase. In older women, this could be due, in part, to the fact that in the life cycle of women they achieve economic stability and establish social and family capital as they age. Furthermore, although they do not have social capital around them, both the legislative body and the institutions offer them different forms of protection. However, in the case of younger women (0–44), inequalities not only persist but tend to increase, particularly in the most disadvantaged groups. This may be due to the fact that women of this age are one of the most vulnerable sectors in times of crisis as they suffer more severely (they or their families, on whom they depend in the case of being minors or not being economically independent), due to unemployment, job insecurity, and various aspects of the so-called feminization of poverty or the intersection between poverty and gender [37]. During childbearing age, childcare can distance them from full inclusion in the labour market or the training necessary for reincorporation when the children have grown up. In the case of single-parent households, they can also bear the double burden of work and the care of children alone. This period, which can last up to two decades, depending on the number of children and the spacing between births, constitutes in itself an element of exclusion for all women, even those of the least deprived levels. In this sense, the risks of death may be related to the mechanisms of stress and frustration-aggression, and although this is shared by women of all classes, it could more sharply affect women from the most disadvantaged DLs.

#### 4.2.2. Amenable Mortality

In general terms, the existence of inequalities by age group, sex, and level of deprivation can be seen. In young men (0–44 years), inequality, practically non-existent in the first period, appears in the second, although it does not reach statistical significance. Regarding the men of intermediate age (45–64) in the first period, a clear gradient of inequality in mortality is perceived, which decreases in the second period. At these ages, paradoxically, unemployment can increase healthy habits (consume less tobacco, alcohol, stress reduction, and sports) and reduce deaths from some amenable causes, such as cardiovascular disease. In the case of the elderly (≥65), an increase in the inequalities towards old age can be perceived from the first to the second period. The combination of the factors mentioned above can influence this age.

In the case of women, inequalities in amenable mortality persist over time. Furthermore, some significant increases in RRs can be seen, i.e., regarding younger women (0–44) in DL5 and women aged 45–64 years in DL4 and DL5. This is consistent with what has been said previously in relation to the all-cause mortality over the life cycle of women. In older women (≥65), inequalities persist with similar gradients in the two periods. This may be because women of these ages do not see their personal economic situation directly affected by the economic downturn as their pensions are not affected, as described above. On the other hand, an increase in the malignant neoplasm of the colon and rectum, as well as malignant neoplasm of cervix uteri is also perceived (see Table A5 of Appendix A). In this combination of simultaneous or successive pro-cyclical and counter-cyclical trends, short or long term, many of the mechanisms of stress, frustration-aggression, or effect budgeting described by Catalano et al. [33] might be at work.

In summary, the patterns of socioeconomic inequality in amenable mortality show some remarkable differences from those of general mortality. In women, the most notable difference occurs in the group over 65 years of age, for which the inequalities in amenable mortality remain over the two periods, whereas inequalities in general mortality are not observed in any of the periods. In the rest of the age groups, amenable mortality is similar to the overall mortality, with inequalities in both periods. In the case of men aged 0–44 years, amenable mortality presents inequalities in the second period that did not exist in the first one, while in overall mortality the inequalities remained over the two periods, although with a slight decrease. In the 45–64-year-old group, inequalities were observed in both amenable and general mortality. Finally, in those over 65 years of age, while inequalities are observed in overall mortality in the second period, the inequalities in amenable mortality were similar in both periods.

### 4.3. Evolution of the Risk of Death

Although both all-cause and amenable mortality have decreased, amenable mortality shows a more pronounced decreasing trend. This pattern had already been described in a similar way in other studies in Europe [23,38,39]. In the Spanish case, this might suggest that the decrease could be due to preventive measures in risk factors and advances in treatments and health technology [6], as well as the entry into force of law 42/2010 on sanitary measures against smoking that regulates the sale, supply, consumption, and advertising of tobacco [34,40].

This decline in all-cause and amenable mortality in times of crisis also seems to corroborate pro-cyclical theories of health. Although this may be so in macro-economic terms, the study of inequalities taking into account both social structure and territory allows us to identify, as in the previous paragraphs, the population groups in which the pro-cyclical decrease in all-cause or amenable mortality is not as pronounced. Furthermore, this is even for the groups in which mortality would have risen, although not significantly, in a counter-cyclical manner, i.e., men older than 65 years, women older than 45 years in the most deprived levels, or women older than 45 years in the level of least deprivation, for all causes; and middle-aged men in low deprivation and high deprivation, and young women in greater deprivation for amenable mortality.

In general terms, as some authors argue, infra-housing, mental disorders, drug addiction, waiting lists, energy poverty, or evictions increase the risks of death [41] and must be analysed at their simultaneous intersection with health [42]. All these processes, present in the cities studied, also validate the counter-cyclical theory. For these reasons, it is important to include inequality in the analysis, and to take into account both pro-cyclical and counter-cyclical trends [11], so that the macro-figure does not hide the reality of the sectors that suffer from the countercyclical trend.

### 4.4. Impact of the Crisis and Hypotheses

Despite the general decrease in amenable mortality, socioeconomic inequalities have remained along the two research periods. This study has been carried out in urban areas of the same region, with common health policy and management, and where access to healthcare was universal during the first period. The start of the crisis meant the widespread application of cuts in healthcare investment, outsourcing of services, exclusion of social sectors from public healthcare, or increased difficulties in accessing it [43].

In this context, the endurance of inequality along the two periods could be due to complex reasons. On the one hand, the impact of health cuts could have affected, to a greater extent, the most disadvantaged population groups, preventing a possible reduction of inequalities. On the other, the results obtained are consistent with other studies carried out in Spain. In them, an effect of the socioeconomic level on mortality was observed independent from that of health care, based on the differences in access to and quality of health care, as previously suggested [6], or the lower participation by the most disadvantaged population in early detection programs (screening programs) of some diseases, such as breast cancer or colon cancer [44,45].

In addition, it should be borne in mind that the prevalence, incidence, and natural course of some diseases could have an effect on amenable mortality and differ between socioeconomic levels, as their risk factors also differ. On the other hand, survival after treatment could be affected by characteristics of individuals related to their socioeconomic level (social support, resources at home, additional medical insurance, etc.), although these variables have not been considered in this study. In any case, amenable mortality proves to be a useful indicator of the degree of efficiency of health systems, also in times of crisis. Failure to reduce or increase amenable mortality is generally accepted as a deterioration of healthcare.

### 4.5. Methodological Strengths and Limitations

This research has the usual limitations of ecological studies. Thus, it is not possible to infer a causal association. The relationship obtained between the DL and the risks of death when using the CTs may not be applicable at the individual level (i.e., ecological fallacy), reflecting both the effect of the individual socioeconomic level and the contextual effect of the area of residence.

The data analysis has been carried out jointly for the three cities. This was mainly due to reasons of statistical power. However, no important differences have been observed among the three cities regarding socioeconomic indicators (Table A2 of Appendix A). In addition, the interactions between the city and the rest of the effects on mortality, such as DL, period, and age, was not significant. Therefore, a differential effect for each city cannot be stated.

Georeferencing often entails difficulties in this kind of research. In our study, the percentage of non-georeferenced deaths is 1.3%, lower than usual, and should have little effect on the results.

The list of amenable causes has been chosen for its potential for comparison with previous studies and also because other lists, even more recent ones, such as that of the AMIEHS project [46], disregards some causes and might not be appropriate for periods such as 2000–2015. The chosen list includes a wide number of amenable causes, sensitive to the effects of austerity and cutbacks in healthcare since the start of the economic crisis in Spain [22].

The inclusion of 50% of deaths from ischemic heart disease could have modified the estimated RRs among the DLs and between periods, as it is a high-frequency cause. To verify this possibility, such RRs were estimated, excluding deaths from this cause. As can be seen in Table A8 and Table A9 of Appendix A, the RRs were hardly modified.

## 5. Conclusions

This study confirms that inequalities persisted during the two study periods, although they have not increased in general terms, except in some sectors, such as young women for amenable mortality. The patterns of inequality evolution showed some differences in amenable mortality and overall mortality in some groups according to sex and age. Thus, while for women of 65 years of age and over inequalities in amenable mortality remained over the two periods, inequalities in overall mortality were not observed in any period. In men, in the group aged 0–44 years, inequalities in amenable mortality were observed in the second period, while in the group aged 65 and over, amenable mortality presented similar inequalities in both periods, while general mortality only in the second period.

At the same time, it has also been found that the evolution of death risks from before the onset of the crisis to the period after the onset presented, overall, a general pro-cyclical trend. However, it has been possible to identify population subgroups by age, sex, and level of deprivation in which the trend, on the contrary, would be counter-cyclical (men older than 65 years, women older than 45 years in the most deprived levels, or women older than 45 years in the level of least deprivation, for all causes; and middle-aged men in low deprivation and high deprivation, as well as young women in greater deprivation for amenable mortality).

The use of the deprivation index has made it possible to identify specific geographic areas with vulnerable populations in all three cities and, at the same time, to identify the change in the level of deprivation (ascending or descending) of the geographical areas throughout the two periods. It is precisely in these areas with the greatest deprivation that more studies that deepen the knowledge of the causes of health inequalities, and those that could indicate the interventions aimed at reducing these inequalities, are needed.

## Figures and Tables

**Table 1:** Descriptive characteristics of the deprivation index according to deprivation levels for the census sections of each city and all three cities.

Town	Deprivation Level (DL) ^a^	Number of Census Tract	2008–2015 (2011 Census)			2000–2007 (2001 Census)		
			Mean	95% CI		Mean	95% CI	
				Lower Limit	Upper Limit		Lower Limit	Upper Limit
Alicante	DL1	17	−0.84	−0.88	−0.79	−0.80	−0.85	−0.76
	DL2	27	−0.56	−0.60	−0.52	−0.52	−0.56	−0.47
	DL3	90	0.00	−0.04	0.04	0.01	−0.03	0.05
	DL4	27	0.44	0.40	0.47	0.42	0.38	0.46
	DL5	17	1.03	0.80	1.25	0.92	0.77	1.06
	Total	178	0.00	−0.08	0.08	0.00	−0.07	0.07
Castellón	DL1	5	−0.55	−0.60	−0.49	−0.67	−0.80	−0.54
	DL2	9	−0.41	−0.45	−0.37	−0.41	−0.45	−0.38
	DL3	30	−0.01	−0.08	0.06	−0.03	−0.10	0.03
	DL4	9	0.41	0.37	0.45	0.47	0.38	0.55
	DL5	5	0.61	0.48	0.74	0.77	0.55	0.98
	Total	58	0.00	−0.10	0.10	0.00	−0.11	0.11
Valencia	DL1	53	−0.72	−0.74	−0.70	−0.78	−0.80	−0.75
	DL2	79	−0.49	−0.51	−0.47	−0.50	−0.52	−0.48
	DL3	266	−0.01	−0.04	0.01	0.00	−0.02	0.03
	DL4	80	0.44	0.42	0.46	0.46	0.44	0.47
	DL5	53	0.85	0.80	0.91	0.82	0.77	0.87
	Total	531	0.00	−0.04	0.04	0.00	−0.04	0.04
All cities together	DL1	75	−0.74	−0.76	−0.71	−0.78	−0.80	−0.75
	DL2	115	−0.50	−0.52	−0.48	−0.50	−0.51	−0.48
	DL3	386	−0.01	−0.03	0.01	0.00	−0.02	0.02
	DL4	116	0.44	0.42	0.45	0.45	0.43	0.46
	DL5	75	0.88	0.81	0.94	0.84	0.79	0.89
	Total	767	0.00	−0.03	0.03	0.00	−0.03	0.03

^a^ DL: Deprivation level of the census tract of residence based on the deprivation index (DI). DL1: DI < P10; DL2: P10 ≤ DI < P25; DL3: P25 ≤ DI < P75; DL4: P75 ≤ DI < P90; DL5: DI ≥ P90. P_q_ = Percentile q.

**Table 2:** Relative risk of death for all causes according to the level of deprivation and 95% confidence intervals (95% CI) specific by age, sex, and period.

Sex	Age	Deprivation Level (DL) ^a^	2000–2007			2008–2015		
			RR	95% CI		RR	95% CI	
				Lower	Upper		Lower	Upper
Men	0–44	DL5	2.034	1.708	2.434	1.582	1.263	1.997
		DL4	1.504	1.269	1.793	1.365	1.097	1.713
		DL3	1.196	1.024	1.406	1.040	0.853	1.282
		DL2	1.168	0.981	1.397	0.881	0.705	1.110
		DL1	1	.	.	1	.	.
	45–64	DL5	1.697	1.525	1.890	1.535	1.369	1.724
		DL4	1.372	1.242	1.519	1.427	1.283	1.591
		DL3	1.168	1.068	1.279	1.146	1.041	1.264
		DL2	1.009	0.911	1.119	0.871	0.781	0.974
		DL1	1	.	.	1	.	.
	≥65	DL5	1.015	0.966	1.066	1.226	1.166	1.289
		DL4	0.963	0.920	1.008	1.135	1.083	1.190
		DL3	0.983	0.945	1.023	1.068	1.025	1.113
		DL2	0.979	0.935	1.025	1.014	0.968	1.062
		DL1	1	.	.	1	.	.
Women	0–44	DL5	1.557	1.202	2.030	1.933	1.401	2.711
		DL4	1.327	1.041	1.706	1.422	1.037	1.984
		DL3	1.142	0.922	1.434	1.294	0.977	1.756
		DL2	1.074	0.841	1.383	1.096	0.802	1.524
		DL1	1	.	.	1	.	.
	45–64	DL5	1.473	1.257	1.727	1.372	1.174	1.606
		DL4	1.262	1.092	1.463	1.198	1.038	1.386
		DL3	1.102	0.971	1.254	0.989	0.873	1.124
		DL2	1.113	0.964	1.287	0.882	0.765	1.018
		DL1	1	.	.	1	.	.
	≥65	DL5	1.000	0.955	1.048	1.010	0.964	1.058
		DL4	1.015	0.972	1.060	0.986	0.945	1.028
		DL3	0.996	0.960	1.034	0.978	0.944	1.014
		DL2	1.087	1.042	1.133	1.024	0.983	1.067
		DL1	1	.	.	1	.	.

Note: ^a^ DL: Deprivation level of the census tract of residence based on the deprivation index (DI). DL1: DI < P10; DL2: P10 ≤ DI < P25; DL3: P25 ≤ DI < P75; DL4: P75 ≤ DI < P90; DL5: DI ≥ P90. P_q_ = Percentile q.

**Table 3:** Relative risk of death for all causes in the 2008–2015 period versus the 2000–2007 period and 95% confidence intervals (95% CI) specific for age, sex, and deprivation level.

Deprivation Level (DL) ^a^	Age	Men			Women		
		RR	95% CI		RR	95% CI	
			Lower	Upper		Lower	Upper
DL1	0–44	0.720	0.562	0.918	0.671	0.470	0.946
	45–64	0.863	0.761	0.977	1.042	0.882	1.232
	≥65	0.829	0.786	0.874	0.993	0.947	1.041
DL2	0–44	0.543	0.467	0.631	0.684	0.556	0.840
	45–64	0.745	0.684	0.811	0.826	0.736	0.927
	≥65	0.859	0.826	0.892	0.935	0.904	0.968
DL3	0–44	0.626	0.577	0.680	0.760	0.678	0.851
	45–64	0.846	0.810	0.885	0.936	0.876	0.999
	≥65	0.901	0.882	0.920	0.975	0.956	0.995
DL4	0–44	0.654	0.568	0.751	0.718	0.582	0.885
	45–64	0.897	0.829	0.970	0.990	0.878	1.115
	≥65	0.977	0.940	1.016	0.964	0.929	1.001
DL5	0-44	0.560	0.479	0.653	0.832	0.657	1.053
	45–64	0.780	0.709	0.859	0.972	0.838	1.127
	≥65	1.002	0.957	1.049	1.002	0.958	1.049

Note: ^a^ DL: Deprivation level for the census tract of residence based on the deprivation index (DI). DL1: DI < P_10_; DL2: P_10_ ≤ DI < P_25_; DL3: P_25_ ≤ DI < P_75_; DL4: P_75_ ≤ DI < P_90_; DL5: DI ≥ P_90_. P_q_ = Percentile q.

**Table 4:** Relative risk of death by amenable causes of death according to deprivation level and 95% confidence intervals (95% CI) specific for age, sex, and period.

Sex	Age	Deprivation Level (DL) ^a^	2000–2007			2008–2015		
			RR	95% CI		RR	95% CI	
				Lower	Upper		Lower	Upper
Men	0–44	DL5	0.997	0.659	1.519	1.256	0.742	2.195
		DL4	0.869	0.593	1.292	1.419	0.876	2.405
		DL3	0.835	0.605	1.183	1.061	0.686	1.736
		DL2	0.979	0.678	1.437	0.884	0.538	1.514
		DL1	1	.	.	1	.	.
	45–64	DL5	2.079	1.607	2.710	1.364	1.046	1.789
		DL4	1.491	1.165	1.925	1.474	1.160	1.891
		DL3	1.345	1.080	1.696	1.049	0.846	1.318
		DL2	1.254	0.979	1.620	0.775	0.602	1.005
		DL1	1	.	.	1	.	.
	≥65	DL5	1.177	0.971	1.429	1.238	0.983	1.564
		DL4	1.090	0.910	1.311	1.297	1.052	1.608
		DL3	1.099	0.939	1.294	1.112	0.925	1.350
		DL2	1.002	0.834	1.209	.960	0.774	1.196
		DL1	1	.	.	1	.	.
Women	0–44	DL5	1.507	0.905	2.564	1.932	1.143	3.413
		DL4	1.673	1.063	2.735	1.405	0.840	2.464
		DL3	1.364	0.908	2.154	1.148	0.727	1.931
		DL2	1.345	0.851	2.205	1.268	0.772	2.195
		DL1	1	.	.	1	.	.
	45–64	DL5	1.347	1.040	1.748	1.696	1.286	2.250
		DL4	1.153	0.912	1.466	1.378	1.064	1.801
		DL3	1.016	0.831	1.254	1.110	0.885	1.411
		DL2	1.109	0.883	1.402	1.033	0.801	1.346
		DL1	1	.	.	1	.	.
	≥65	DL5	1.665	1.338	2.083	1.665	1.284	2.173
		DL4	1.384	1.118	1.723	1.394	1.089	1.798
		DL3	1.251	1.037	1.523	1.367	1.102	1.718
		DL2	1.062	0.853	1.330	1.309	1.026	1.684
		DL1	1	.	.	1	.	.

Note: ^a^ DL: Deprivation level of the census tract of residence based on the deprivation index (DI). DL1: DI < P_10_; DL2: P_10_ ≤ DI < P_25_; DL3: P_25_ ≤ DI < P_75_; DL4: P_75_ ≤ DI < P_90_; DL5: DI ≥ P_90._ P_q_ = Percentile q.

**Table 5:** Relative risk of death for amenable causes of death in the 2008–2015 period versus the 2000–2007 period and 95% confidence intervals (95% CI) specific by age, sex, and deprivation level.

Deprivation Level (DL) ^a^	Age	Men			Women		
		RR	95% CI		RR	95% CI	
			Lower	Upper		Lower	Upper
DL1	0–44	0.598	0.343	1.010	0.953	0.508	1.761
	45–64	1.060	0.786	1.429	0.765	0.571	1.021
	≥65	0.640	0.507	0.806	0.681	0.516	0.896
DL2	0–44	0.540	0.382	0.757	0.899	0.641	1.260
	45–64	0.655	0.536	0.799	0.713	0.589	0.863
	≥65	0.613	0.518	0.724	0.839	0.697	1.011
DL3	0–44	0.760	0.626	0.921	0.802	0.656	0.980
	45–64	0.827	0.744	0.918	0.836	0.748	0.935
	≥65	0.648	0.593	0.708	0.745	0.674	0.823
DL4	0–44	0.976	0.695	1.370	0.801	0.559	1.141
	45–64	1.048	0.873	1.258	0.914	0.746	1.121
	≥65	0.761	0.650	0.890	0.686	0.571	0.823
DL5	0–44	0.753	0.493	1.142	1.223	0.799	1.884
	45–64	0.695	0.554	0.871	0.963	0.752	1.234
	≥65	0.673	0.554	0.816	0.681	0.555	0.835

Note: ^a^ DL: Deprivation level for the census tract of residence based on the deprivation index (DI). DL1: DI < P_10_; DL2: P_10_ ≤ DI < P_25_; DL3: P_25_ ≤ DI < P_75_; DL4: P_75_ ≤ DI < P_90_; DL5: DI ≥ P_90_. P_q_ = Percentile q.

## Appendix A

**Table A1:** International Classification of Diseases Codes, 10th revision (ICD-10), and the age ranges for the amenable causes.

	Amenable Causes	ICD-10	Age
1	Intestinal infections	A00-09	0–14
2	Tuberculosis	A15-A19, B90	0–74
3	Other infections (diphtheria, tetanus, poliomyelitis)	A36, A35, A80	0–74
4	Whooping cough	A37	0–14
5	Septicaemia	A40-A41	0–74
6	Measles	B05	1–14
7	Malignant neoplasm of colon and rectum	C18-C21	0–74
8	Malignant neoplasm of skin	C44	0–74
9	Malignant neoplasm of breast	C50	0–74
10	Malignant neoplasm of cervix uteri	C53	0–74
11	Malignant neoplasm of cervix uteri and body of uterus	C54-C55	0–44
12	Malignant neoplasm of testis	C62	0–74
13	Hodgkin’s disease	C81	0–74
14	Leukaemia	C91-C95	0–44
15	Diseases of the thyroid	E00-E07	0–74
16	Diabetes mellitus	E10-E14	0–49
17	Epilepsy	G40-G41	0–74
18	Chronic rheumatic heart disease	I05-I09	0–74
19	Hypertensive disease	I10-I13, I15	0–74
20	Ischaemic heart disease (50% of deaths)	I20-I25	0–74
21	Cerebrovascular disease	I60-I69	0–74
22	All respiratory diseases (excluding pneumonia and influenza)	J00-J09, J20-J99	1–14
23	Influenza	J10-J11	0–74
24	Pneumonia	J12-J18	0–74
25	Peptic ulcer	K25-K27	0–74
26	Appendicitis	K35-K38	0–74
27	Abdominal hernia	K40-K46	0–74
28	Cholelithiasis and cholecystitis	K80-K81	0–74
29	Nephritis and nephrosis	N00-N07,N17-N19,N25-N27	0–74
30	Benign prostatic hyperplasia	N40	0–74
31	Maternal death	O00-O99	All
32	Congenital cardiovascular anomalies	Q20-Q28	0–74
33	Perinatal deaths, all causes	P00-P96, A33, A34	All
34	Misadventures to patients during surgical and medical care	Y60-Y69, Y83-Y84	All

**Table A2:** Average values of the socioeconomic indicators by city, period, and percentile-based classification of the deprivation index.

Socioeconomic Indicator	Deprivation Level (DL) ^a^	Valencia				Alicante				Castellón			
		2000–2007 (2001 Census)		2008–2015 (2011 Census)		2000–2007 (2001 Census)		2008–2015 (2011 Census)		2000–2007 (2001 Census)		2008–2015 (2011 Census)	
		Mean	Std. Dev.	Mean	Std. Dev.	Mean	Std. Dev.	Mean	Std. Dev.	Mean	Std. Dev.	Mean	Std. Dev.
People aged 16 or over who have a manual job	DL1	0.177	0.034	0.142	0.028	0.230	0.044	0.174	0.045	0.313	0.034	0.276	0.015
	DL2	0.276	0.045	0.231	0.040	0.332	0.052	0.298	0.060	0.420	0.044	0.349	0.037
	DL3	0.479	0.087	0.444	0.090	0.546	0.087	0.527	0.101	0.575	0.085	0.529	0.087
	DL4	0.647	0.045	0.605	0.055	0.686	0.061	0.697	0.066	0.751	0.033	0.685	0.029
	DL5	0.710	0.055	0.696	0.063	0.797	0.043	0.786	0.075	0.798	0.048	0.726	0.051
	Total	0.467	0.172	0.432	0.177	0.529	0.174	0.509	0.194	0.575	0.153	0.521	0.149
People aged over 16 years out of work	DL1	0.099	0.019	0.204	0.034	0.096	0.019	0.220	0.033	0.086	0.012	0.283	0.033
	DL2	0.122	0.024	0.233	0.031	0.115	0.024	0.262	0.035	0.088	0.017	0.308	0.034
	DL3	0.146	0.027	0.287	0.042	0.138	0.035	0.345	0.051	0.095	0.015	0.338	0.050
	DL4	0.162	0.030	0.333	0.042	0.155	0.044	0.399	0.038	0.095	0.014	0.405	0.051
	DL5	0.200	0.046	0.388	0.053	0.197	0.043	0.502	0.082	0.112	0.010	0.369	0.017
	Total	0.145	0.038	0.287	0.064	0.138	0.042	0.343	0.088	0.095	0.016	0.342	0.056
People aged 16 or over in temporary employment	DL1	0.160	0.023	0.111	0.030	0.182	0.030	0.125	0.016	0.171	0.031	0.137	0.022
	DL2	0.191	0.028	0.131	0.037	0.213	0.029	0.153	0.024	0.212	0.021	0.156	0.013
	DL3	0.238	0.036	0.167	0.045	0.280	0.046	0.199	0.030	0.232	0.033	0.182	0.034
	DL4	0.275	0.036	0.211	0.060	0.352	0.046	0.243	0.039	0.242	0.043	0.203	0.020
	DL5	0.326	0.042	0.239	0.063	0.414	0.086	0.302	0.048	0.286	0.047	0.189	0.033
	Total	0.238	0.056	0.170	0.060	0.284	0.080	0.201	0.056	0.230	0.042	0.178	0.033
People aged over 16 years with low education level	DL1	0.112	0.033	0.076	0.020	0.126	0.030	0.088	0.029	0.204	0.036	0.126	0.012
	DL2	0.187	0.034	0.120	0.023	0.213	0.042	0.128	0.037	0.253	0.024	0.148	0.012
	DL3	0.301	0.060	0.198	0.045	0.332	0.058	0.222	0.042	0.354	0.054	0.207	0.045
	DL4	0.417	0.040	0.280	0.036	0.419	0.043	0.293	0.045	0.496	0.056	0.279	0.028
	DL5	0.517	0.051	0.353	0.047	0.556	0.073	0.410	0.099	0.576	0.051	0.363	0.046
	Total	0.304	0.121	0.202	0.086	0.329	0.123	0.223	0.098	0.366	0.115	0.215	0.073
People aged 16 to 29 years with low education level	DL1	0.030	0.014	0.018	0.008	0.049	0.023	0.019	0.008	0.070	0.025	0.061	0.029
	DL2	0.051	0.017	0.033	0.021	0.073	0.024	0.033	0.018	0.100	0.028	0.051	0.014
	DL3	0.092	0.025	0.056	0.031	0.114	0.040	0.083	0.035	0.136	0.028	0.088	0.030
	DL4	0.139	0.028	0.103	0.049	0.174	0.037	0.128	0.046	0.226	0.052	0.138	0.030
	DL5	0.226	0.058	0.197	0.073	0.287	0.105	0.303	0.161	0.323	0.046	0.188	0.013
	Total	0.100	0.059	0.070	0.061	0.127	0.078	0.097	0.094	0.155	0.075	0.096	0.047

Note: ^a^ DL: Deprivation level of the census track of residence based on the deprivation index (DI). DL1: DI < P_10_; DL2: P_10_ ≤ DI < P_25_; DL3: P_25_ ≤ DI < P_75_; DL4: P_75_ ≤ DI < P_90_; DL5: DI ≥ P_90_; P_q_ = Percentile q.

**Table A3:** Average annual population for the three cities by period, age group, sex, and percentile-based classification of the deprivation index.

Period	Deprivation Level (DL) ^a^	0–44		45–64		≥65	
		Men	Women	Men	Women	Men	Women
2000–2007	DL1	27,649	28,172	11,642	14,050	6802	11,100
	DL2	61,455	61,018	23,269	26,272	12,625	19,926
	DL3	198,787	193,125	71,408	79,503	41,207	62,468
	DL4	55,670	51,990	20,026	21,407	12,805	18,065
	DL5	34,655	30,895	11,070	11,989	8705	12,994
	Total	378,213	365,200	137,416	153,219	82,144	124,552
2008–2015	DL1	23,122	23,128	11,377	13,725	7600	12,044
	DL2	69,293	68,923	29,524	33,207	15,390	22,695
	DL3	198,369	191,465	85,647	94,619	47,885	70,375
	DL4	52,128	48,464	21,396	22,664	13,292	19,839
	DL5	35,897	31,253	12,886	12,951	8486	12,493
	Total	378,808	363,229	160,829	177,164	92,650	137,443

Note: ^a^ DL: Deprivation level of the census track of residence based on the deprivation index (DI). DL1: DI < P_10_; DL2: P_10_ ≤ DI < P_25_; DL3: P_25_ ≤ DI < P_75_; DL4: P_75_ ≤ DI < P_90_; DL5: DI ≥ P_90_. P_q_ = Percentile q.

**Table A4:** Frequencies and percentages ^a^ of death for various amenable causes, by sex, period, and level of deprivation (DL) ^b^. All cities together, 2000–2015.

Men	Deprivation Level (DL) ^b^											
	DL1		DL2		DL3		DL4		DL5		Total	
	2000–2007	2008–2015	2000–2007	2008–2015	2000–2007	2008–2015	2000–2007	2008–2015	2000–2007	2008–2015	2000–2007	2008–2015
Septicaemia	12	3	20	12	74	45	20	16	25	6	151	82
	4.0%	1.3%	3.2%	2.6%	3.5%	2.6%	3.1%	2.7%	5.2%	1.7%	3.7%	2.4%
Malignant neoplasm of colon and rectum	73	70	135	108	416	448	126	144	104	85	854	855
	24.6%	30.3%	21.8%	23.0%	19.9%	25.8%	19.7%	24.5%	21.8%	24.6%	20.7%	25.4%
Malignant neoplasm of breast	2	0	2	0	1	5	0	2	0	0	5	7
	0.7%	0.0%	0.3%	0.0%	0.0%	0.3%	0.0%	0.3%	0.0%	0.0%	0.1%	0.2%
Chronic rheumatic heart disease	0	2	9	8	31	32	10	5	8	2	58	49
	0.0%	0.9%	1.5%	1.7%	1.5%	1.8%	1.6%	0.9%	1.7%	0.6%	1.4%	1.5%
Hypertensive disease	9	6	11	25	59	74	19	26	8	17	106	148
	3.0%	2.6%	1.8%	5.3%	2.8%	4.3%	3.0%	4.4%	1.7%	4.9%	2.6%	4.4%
Ischaemic heart disease (50% of deaths)	86	56	175	128	606	446	180	143	117	91	1164	864
	29.0%	24.2%	28.2%	27.3%	29.1%	25.6%	28.1%	24.4%	24.5%	26.4%	28.2%	25.6%
Cerebrovascular disease	63	47	128	89	437	314	127	105	105	65	860	620
	21.2%	20.3%	20.6%	19.0%	20.9%	18.1%	19.8%	17.9%	22.0%	18.8%	20.9%	18.4%
All respiratory diseases (excl. pneumonia and influenza)	13	21	47	25	157	126	73	48	41	31	331	251
	4.4%	9.1%	7.6%	5.3%	7.5%	7.2%	11.4%	8.2%	8.6%	9.0%	8.0%	7.4%
Pneumonia	10	5	16	21	90	62	28	25	18	14	162	127
	3.4%	2.2%	2.6%	4.5%	4.3%	3.6%	4.4%	4.3%	3.8%	4.1%	3.9%	3.8%
Perinatal deaths, all causes	9	5	25	18	56	44	12	14	13	10	115	91
	3.0%	2.2%	4.0%	3.8%	2.7%	2.5%	1.9%	2.4%	2.7%	2.9%	2.8%	2.7%
Misadventures to patients during surgical and medical care	0	2	2	5	1	19	1	6	1	2	5	34
	0.0%	0.9%	0.3%	1.1%	0.0%	1.1%	0.2%	1.0%	0.2%	0.6%	0.1%	1.0%
Other amenable causes	20	14	50	30	158	124	45	53	37	22	310	243
	6.7%	6.1%	8.1%	6.4%	7.6%	7.1%	7.0%	9.0%	7.8%	6.4%	7.5%	7.2%
Total amenable	297	231	620	469	2086	1739	641	587	477	345	4121	3371
**Women**	**Deprivation Level (DL) ^b^**											
	**DL1**		**DL2**		**DL3**		**DL4**		**DL5**		Total	
	**2000–2007**	**2008–2015**	**2000–2007**	**2008–2015**	**2000–2007**	**2008–2015**	**2000–2007**	**2008–2015**	**2000–2007**	**2008–2015**	2000–2007	2008–2015
Septicaemia	7	6	8	6	36	29	10	7	13	10	74	58
	2.8%	3.2%	1.6%	1.2%	2.2%	2.0%	1.9%	1.6%	3.3%	3.1%	1.9%	2.0%
Malignant neoplasm of colon and rectum	44	33	86	91	260	280	81	76	52	70	523	550
	17.7%	17.7%	16.7%	18.8%	15.6%	18.8%	15.4%	17.4%	13.2%	21.4%	15.6%	18.8%
Malignant neoplasm of breast	86	64	173	173	431	419	138	119	90	57	918	832
	34.5%	34.4%	33.5%	35.7%	25.8%	28.2%	26.2%	27.2%	22.9%	17.4%	27.4%	28.5%
Malignant neoplasm of cervix uteri or cervix uteri and body of uterus	15	11	34	40	128	162	38	48	34	33	249	294
	6.0%	5.9%	6.6%	8.2%	7.7%	10.9%	7.2%	11.0%	8.7%	10.1%	7.4%	10.1%
Chronic rheumatic heart disease	6	12	17	11	64	42	27	11	15	13	129	89
	2.4%	6.5%	3.3%	2.3%	3.8%	2.8%	5.1%	2.5%	3.8%	4.0%	3.8%	3.0%
Hypertensive disease	3	7	4	11	37	49	23	13	11	11	78	91
	1.2%	3.8%	0.8%	2.3%	2.2%	3.3%	4.4%	3.0%	2.8%	3.4%	2.3%	3.1%
Ischaemic heart disease (50% of deaths)	21	10	39	25	135	62	37	30	36	20	268	147
	8.4%	5.4%	7.6%	5.2%	8.1%	4.2%	7.0%	6.9%	9.2%	6.1%	8.0%	5.0%
Cerebrovascular disease	41	23	86	53	309	200	96	63	74	58	606	397
	16.5%	12.4%	16.7%	10.9%	18.5%	13.5%	18.2%	14.4%	18.8%	17.7%	18.1%	13.6%
All respiratory diseases (excl. pneumonia and influenza)	7	4	16	14	72	69	17	20	17	14	129	121
	2.8%	2.2%	3.1%	2.9%	4.3%	4.6%	3.2%	4.6%	4.3%	4.3%	3.8%	4.1%
Pneumonia	4	6	12	11	45	34	17	7	18	8	96	66
	1.6%	3.2%	2.3%	2.3%	2.7%	2.3%	3.2%	1.6%	4.6%	2.4%	2.9%	2.3%
Perinatal deaths, all causes	5	4	18	18	41	36	8	17	7	10	79	85
	2.0%	2.2%	3.5%	3.7%	2.5%	2.4%	1.5%	3.9%	1.8%	3.1%	2.4%	2.9%
Misadventures to patients during surgical and medical care	1	0	0	8	3	17	1	6	0	2	5	33
	0.4%	0.0%	0.0%	1.6%	0.2%	1.1%	0.2%	1.4%	0.0%	0.6%	0.1%	1.1%
Other amenable causes	9	6	23	24	107	87	34	20	24	21	199	158
	3.6%	3.2%	4.5%	4.9%	6.4%	5.9%	6.5%	4.6%	6.1%	6.4%	5.9%	5.4%
Total amenable	249	186	516	485	1668	1486	527	437	393	327	3353	2921

Note: ^a^ Percentages have been calculated in relation to the total of amenable deaths for the period and DL. ^b^ DL: Deprivation level of the census track of residence based on the deprivation index (DI). DL1: DI < P_10_; DL2: P_10_ ≤ DI < P_25_; DL3: P_25_ ≤ DI < P_75_; DL4: P_75_ ≤ DI < P_90_; DL5: DI ≥ P_90_. P_q_ = Percentile q.

**Table A5:** Frequencies and percentages of death according to the large groups of the ICD-10, by sex, level of deprivation, and period. All cities together, 2000–2015.

Men	Deprivation Level (DL) ^a^												Total
	DL1		DL2		DL3		DL4		DL5		Total		
	2000–2007	2008–2015	2000–2007	2008–2015	2000–2007	2008–2015	2000–2007	2008–2015	2000–2007	2008–2015	2000–2007	2008–2015	
I: Infectious and parasitic diseases	60	37	154	124	508	404	192	133	200	108	1114	806	1920
	1.7%	1.2%	2.3%	1.9%	2.3%	1.8%	2.8%	2.0%	4.0%	2.3%	2.5%	1.8%	2.2%
II: Neoplasms	1208	1036	2163	2277	7214	7812	2252	2244	1587	1577	14,424	14,946	29,370
	34.0%	32.5%	32.3%	34.0%	32.4%	34.6%	32.5%	33.2%	31.8%	34.0%	32.5%	34.1%	33.3%
III: Diseases of the blood, and inmunity disorders	8	10	18	25	60	56	24	21	9	11	119	123	242
	0.2%	0.3%	0.3%	0.4%	0.3%	0.2%	0.3%	0.3%	0.2%	0.2%	0.3%	0.3%	0.3%
IV: Endocrine, nutritional and metabolic diseases	82	68	154	150	492	518	174	181	123	119	1025	1036	2061
	2.3%	2.1%	2.3%	2.2%	2.2%	2.3%	2.5%	2.7%	2.5%	2.6%	2.3%	2.4%	2.3%
V: Mental and behavioural disorders	66	80	147	181	435	659	145	190	95	130	888	1240	2128
	1.9%	2.5%	2.2%	2.7%	2.0%	2.9%	2.1%	2.8%	1.9%	2.8%	2.0%	2.8%	2.4%
VI-VIII: Diseases of the nervous system and organ senses	123	161	195	367	718	1075	216	284	130	188	1382	2075	3457
	3.5%	5.1%	2.9%	5.5%	3.2%	4.8%	3.1%	4.2%	2.6%	4.0%	3.1%	4.7%	3.9%
IX: Diseases of the circulatory system	1129	977	2098	1901	6775	6316	1942	1869	1376	1215	13,320	12,278	25,598
	31.8%	30.7%	31.4%	28.4%	30.5%	28.0%	28.0%	27.6%	27.6%	26.2%	30.0%	28.0%	29.0%
X: Diseases of the respiratory system	386	401	810	761	2796	2675	940	842	685	627	5617	5306	10,923
	10.9%	12.6%	12.1%	11.4%	12.6%	11.9%	13.6%	12.5%	13.7%	13.5%	12.6%	12.1%	12.4%
XI: Diseases of the digestive system	161	148	280	309	1195	1130	400	377	294	251	2330	2215	4545
	4.5%	4.6%	4.2%	4.6%	5.4%	5.0%	5.8%	5.6%	5.9%	5.4%	5.2%	5.1%	5.1%
XII: Diseases of the skin and subcutaneous tissue	10	7	13	8	48	49	9	19	6	12	86	95	181
	0.3%	0.2%	0.2%	0.1%	0.2%	0.2%	0.1%	0.3%	0.1%	0.3%	0.2%	0.2%	0.2%
XIII: Diseases of the musculoskeletal system and connective tissue	12	15	30	34	71	90	30	32	18	21	161	192	353
	0.3%	0.5%	0.4%	0.5%	0.3%	0.4%	0.4%	0.5%	0.4%	0.5%	0.4%	0.4%	0.4%
XIV: Diseases of the genitourinary system	102	99	156	201	509	560	154	197	119	106	1040	1163	2203
	2.9%	3.1%	2.3%	3.0%	2.3%	2.5%	2.2%	2.9%	2.4%	2.3%	2.3%	2.7%	2.5%
XV: Pregnancy, childbirth and the puerperium	9	5	25	18	56	44	12	14	13	10	115	91	206
	0.3%	0.2%	0.4%	0.3%	0.3%	0.2%	0.2%	0.2%	0.3%	0.2%	0.3%	0.2%	0.2%
XVI: Certain conditions originating in the perinatal period	5	7	23	15	48	40	13	16	10	11	99	89	188
	0.1%	0.2%	0.3%	0.2%	0.2%	0.2%	0.2%	0.2%	0.2%	0.2%	0.2%	0.2%	0.2%
XVII: Congenital malformations	38	25	78	72	242	231	70	55	56	53	484	436	920
	1.1%	0.8%	1.2%	1.1%	1.1%	1.0%	1.0%	0.8%	1.1%	1.1%	1.1%	1.0%	1.0%
XVIII: Symptoms and signs not elsewhere classified	151	111	347	254	1073	893	364	287	273	206	2208	1751	3959
	4.3%	3.5%	5.2%	3.8%	4.8%	4.0%	5.2%	4.2%	5.5%	4.4%	5.0%	4.0%	4.5%
Total	3550	3187	6691	6697	22,240	22,552	6937	6761	4994	4645	44,412	43,842	88,254
	100.0%	100.0%	100.0%	100.0%	100.0%	100.0%	100.0%	100.0%	100.0%	100.0%	100.0%	100.0%	100.0%
**Women**	**Deprivation Level (DL) ^a^**												**Total**
	**DL1**		**DL2**		**DL3**		**DL4**		**DL5**		**Total**		
	**2000–2007**	**2008–2015**	**2000–2007**	**2008–2015**	**2000–2007**	**2008–2015**	**2000–2007**	**2008–2015**	**2000–2007**	**2008–2015**	**2000–2007**	**2008–2015**	
I: Infectious and parasitic diseases	72	63	112	116	378	385	122	104	110	117	794	785	1579
	2.0%	1.6%	1.6%	1.5%	1.8%	1.7%	2.0%	1.6%	2.5%	2.8%	1.9%	1.8%	1.8%
II: Neoplasms	813	849	1610	1763	4504	5201	1339	1502	953	963	9219	10,278	19,497
	22.3%	22.0%	22.5%	23.3%	21.7%	23.0%	21.8%	23.4%	22.0%	23.0%	21.9%	23.0%	22.5%
III: Diseases of the blood, and inmunity disorders	14	20	26	40	98	95	20	32	21	23	179	210	389
	0.4%	0.5%	0.4%	0.5%	0.5%	0.4%	0.3%	0.5%	0.5%	0.5%	0.4%	0.5%	0.4%
IV: Endocrine, nutritional and metabolic diseases	104	104	251	224	737	798	271	242	183	170	1546	1538	3084
	2.9%	2.7%	3.5%	3.0%	3.5%	3.5%	4.4%	3.8%	4.2%	4.1%	3.7%	3.4%	3.6%
V: Mental and behavioural disorders	134	214	346	478	902	1249	239	358	163	255	1784	2554	4338
	3.7%	5.5%	4.8%	6.3%	4.3%	5.5%	3.9%	5.6%	3.8%	6.1%	4.2%	5.7%	5.0%
VI-VIII: Diseases of the nervous system and organ senses	184	303	351	636	1068	1793	310	485	205	314	2118	3531	5649
	5.1%	7.9%	4.9%	8.4%	5.1%	7.9%	5.0%	7.5%	4.7%	7.5%	5.0%	7.9%	6.5%
IX: Diseases of the circulatory system	1474	1439	2861	2600	8171	7840	2403	2214	1642	1469	16,551	15,562	32,113
	40.5%	37.3%	39.9%	34.4%	39.3%	34.7%	39.0%	34.4%	37.9%	35.1%	39.3%	34.9%	37.0%
X: Diseases of the respiratory system	351	360	661	700	2080	2092	593	572	403	370	4088	4094	8182
	9.6%	9.3%	9.2%	9.3%	10.0%	9.3%	9.6%	8.9%	9.3%	8.8%	9.7%	9.2%	9.4%
XI: Diseases of the digestive system	188	148	359	298	1106	1056	352	335	269	185	2274	2022	4296
	5.2%	3.8%	5.0%	3.9%	5.3%	4.7%	5.7%	5.2%	6.2%	4.4%	5.4%	4.5%	5.0%
XII: Diseases of the skin and subcutaneous tissue	13	22	24	37	94	129	35	40	22	14	188	242	430
	0.4%	0.6%	0.3%	0.5%	0.5%	0.6%	0.6%	0.6%	0.5%	0.3%	0.4%	0.5%	0.5%
XIII: Diseases of the musculoskeletal system and connective tissue	28	38	70	75	182	215	66	69	50	31	396	428	824
	0.8%	1.0%	1.0%	1.0%	0.9%	1.0%	1.1%	1.1%	1.2%	0.7%	0.9%	1.0%	1.0%
XIV: Diseases of the genitourinary system	114	134	217	270	607	837	166	227	141	136	1245	1604	2849
	3.1%	3.5%	3.0%	3.6%	2.9%	3.7%	2.7%	3.5%	3.3%	3.2%	3.0%	3.6%	3.3%
XV: Pregnancy, childbirth and the puerperium	0	0	0	2	2	3	1	1	0	0	3	6	9
	0.0%	0.0%	0.0%	0.0%	0.0%	0.0%	0.0%	0.0%	0.0%	0.0%	0.0%	0.0%	0.0%
XVI: Certain conditions originating in the perinatal period	5	4	18	18	41	36	8	17	7	10	79	85	164
	0.1%	0.1%	0.3%	0.2%	0.2%	0.2%	0.1%	0.3%	0.2%	0.2%	0.2%	0.2%	0.2%
XVII: Congenital malformations	7	4	16	16	61	41	10	15	7	5	101	81	182
	0.2%	0.1%	0.2%	0.2%	0.3%	0.2%	0.2%	0.2%	0.2%	0.1%	0.2%	0.2%	0.2%
XVIII: Symptoms and signs not elsewhere classified	58	77	104	127	256	259	84	68	44	48	546	579	1125
	1.6%	2.0%	1.5%	1.7%	1.2%	1.1%	1.4%	1.1%	1.0%	1.1%	1.3%	1.3%	1.3%
XX: External causes of morbidity and mortality	80	79	142	167	485	561	137	146	112	76	956	1029	1985
	2.2%	2.0%	2.0%	2.2%	2.3%	2.5%	2.2%	2.3%	2.6%	1.8%	2.3%	2.3%	2.3%
Total	3639	3858	7168	7567	20,772	22,590	6156	6427	4332	4186	42,067	44,628	86,695
	100.0%	100.0%	100.0%	100.0%	100.0%	100.0%	100.0%	100.0%	100.0%	100.0%	100.0%	100.0%	100.0%

Note: ^a^ DL: Deprivation level of the census track of residence based on the deprivation index (DI). DL1: DI < P_10_; DL2: P_10_ ≤ DI < P_25_; DL3: P_25_ ≤ DI < P_75_; DL4: P_75_ ≤ DI < P_90_; DL5: DI ≥ P_90_. P_q_ = Percentile q.

**Table A6:** Mortality rates (×100,000) for all causes, by sex, age group, deprivation level, and period under study. All cities together.

Sex	Age	Deprivation Level (DL) ^a^	Period	
			2000–2007	2008–2015
Men	0–44	DL1	77.3	55.7
		DL2	90.3	49.1
		DL3	92.4	57.9
		DL4	116.3	76.0
		DL5	157.3	88.1
	45–64	DL1	580.8	501.0
		DL2	586.1	436.5
		DL3	678.1	574.0
		DL4	797.1	715.1
		DL5	985.8	769.3
	≥65	DL1	5215.2	4324.3
		DL2	5105.4	4383.7
		DL3	5125.3	4617.4
		DL4	5021.5	4908.0
		DL5	5291.3	5301.7
Women	0–44	DL1	39.5	26.5
		DL2	42.4	29.0
		DL3	45.1	34.3
		DL4	52.4	37.7
		DL5	61.5	51.2
	45–64	DL1	242.9	253.2
		DL2	270.2	223.2
		DL3	267.6	250.3
		DL4	306.6	303.4
		DL5	357.7	347.5
	≥65	DL1	3690.7	3663.7
		DL2	4010.4	3751.5
		DL3	3676.7	3584.8
		DL4	3745.6	3611.7
		DL5	3691.3	3700.3

Note: ^a^ DL: Deprivation level of the census track of residence based on the deprivation index (DI). DL1: DI < P_10_; DL2: P_10_ ≤ DI < P_25_; DL3: P_25_ ≤ DI < P_75_; DL4: P_75_ ≤ DI < P_90_; DL5: DI ≥ P_90_. P_q_ = Percentile q.

**Table A7:** Mortality rates (×100,000) by susceptible causes, sex, age group, level of deprivation, and period of study. All cities together.

Sex	Age	Deprivation Level (DL) ^a^	Period	
			2000–2007	2008–2015
Men	0–44	DL1	18.1	10.8
		DL2	17.7	9.6
		DL3	15.1	11.5
		DL4	15.7	15.3
		DL5	18.0	13.6
	45–64	DL1	91.3	96.7
		DL2	114.4	74.9
		DL3	122.7	101.4
		DL4	136.1	142.6
		DL5	189.7	131.9
	≥65	DL1	316.1	202.3
		DL2	316.9	194.1
		DL3	347.3	225.0
		DL4	344.6	262.4
		DL5	371.9	250.4
Women	0–44	DL1	10.2	9.7
		DL2	13.7	12.3
		DL3	13.9	11.2
		DL4	17.1	13.7
		DL5	15.4	18.8
	45–64	DL1	95.2	72.9
		DL2	105.6	75.3
		DL3	96.7	80.9
		DL4	109.8	100.4
		DL5	128.3	123.5
	≥65	DL1	134.0	91.3
		DL2	142.4	119.5
		DL3	167.7	124.9
		DL4	185.4	127.3
		DL5	223.2	152.1

Note: ^a^ DL: Deprivation level of the census track of residence based on the deprivation index (DI). DL1: DI < P_10_; DL2: P_10_ ≤ DI < P_25_; DL3: P_25_ ≤ DI < P_75_; DL4: P_75_ ≤ DI < P_90_; DL5: DI ≥ P_90_. P_q_ = Percentile q.

**Table A8:** Relative risks of death by amenable causes of death (excluding ischemic heart disease) according to deprivation level and 95% confidence intervals (95% CI) by age, sex, and period. All cities together.

Sex	Age	Deprivation Level (DL) ^a^	2000–2007			2008–2015		
			RR	95% CI		RR	95% CI	
				Lower	Upper		Lower	Upper
Men	0–44	DL5	1.013	0.660	1.568	1.369	0.768	1.369
		DL4	0.765	0.508	1.166	1.525	0.896	1.525
		DL3	0.789	0.564	1.137	1.129	0.697	1.129
		DL2	0.961	0.655	1.434	0.959	0.555	0.959
		DL1	1.000			1.000		
	45–64	DL5	2.270	1.667	3.128	1.324	0.953	1.324
		DL4	1.499	1.111	2.051	1.586	1.186	1.586
		DL3	1.333	1.022	1.773	1.106	0.850	1.106
		DL2	1.290	0.956	1.765	0.784	0.575	0.784
		DL1	1.000			1.000		
	≥65	DL5	1.269	1.010	1.601	1.179	0.912	1.179
		DL4	1.167	0.940	1.457	1.223	0.968	1.223
		DL3	1.134	0.939	1.384	1.029	0.839	1.029
		DL2	1.009	0.808	1.266	0.865	0.679	0.865
		DL1	1.000			1.000		
Women	0–44	DL5	1.534	0.913	2.639	2.014	1.196	3.549
		DL4	1.675	1.054	2.770	1.379	0.823	2.420
		DL3	1.426	0.942	2.274	1.141	0.723	1.920
		DL2	1.490	0.940	2.459	1.230	0.747	2.134
		DL1	1.000			1.000		
	45–64	DL5	1.387	1.061	1.818	1.521	1.155	2.012
		DL4	1.205	0.945	1.547	1.218	0.942	1.587
		DL3	1.042	0.846	1.298	1.003	0.804	1.268
		DL2	1.102	0.869	1.408	0.958	0.746	1.241
		DL1	1.000			1.000		
	≥65	DL5	1.613	1.281	2.044	1.796	1.356	2.398
		DL4	1.377	1.101	1.733	1.530	1.173	2.018
		DL3	1.217	1.000	1.498	1.561	1.235	2.004
		DL2	1.052	0.835	1.333	1.432	1.100	1.885
		DL1	1.000			1.000		

Note: ^a^ DL: Deprivation level of the census track of residence based on the deprivation index (DI). DL1: DI < P_10_; DL2: P_10_ ≤ DI < P_25_; DL3: P_25_ ≤ DI < P_75_; DL4: P_75_ ≤ DI < P_90_; DL5: DI ≥ P_90_. P_q_ = Percentile q.

**Table A9:** Relative risks of death by amenable causes (excluding ischemic heart disease) for the period 2008–2015 as compared to the period 2000–2007 and 95% confidence intervals (95% CI) by age, sex, and deprivation level. All cities together.

Deprivation Level (DL) ^a^	Age	Men			Women		
		RR	95% CI		RR	95% CI	
			Lower	Upper		Lower	Upper
DL1	0–44	0.517	0.280	0.912	0.997	0.528	1.855
	45–64	1.041	0.722	1.503	0.888	0.663	1.187
	≥65	0.773	0.591	1.008	0.623	0.461	0.836
DL2	0–44	0.516	0.357	0.739	0.823	0.588	1.151
	45–64	0.633	0.496	0.805	0.772	0.634	0.939
	≥65	0.663	0.543	0.808	0.848	0.697	1.031
DL3	0–44	0.740	0.600	0.909	0.798	0.652	0.975
	45–64	0.864	0.761	0.982	0.855	0.762	0.959
	≥65	0.701	0.632	0.777	0.799	0.719	0.887
DL4	0–44	1.030	0.710	1.494	0.820	0.570	1.175
	45–64	1.102	0.885	1.374	0.897	0.728	1.106
	≥65	0.810	0.675	0.970	0.692	0.571	0.838
DL5	0–44	0.698	0.446	1.082	1.309	0.857	2.018
	45–64	0.608	0.461	0.798	0.974	0.755	1.256
	≥65	0.718	0.574	0.895	0.693	0.557	0.860

Note: ^a^ DL: Deprivation level for the census track of residence based on the deprivation index (DI). DL1: DI < P_10_; DL2: P_10_ ≤ DI < P_25_; DL3: P_25_ ≤ DI < P_75_; DL4: P_75_ ≤ DI < P_90_; DL5: DI ≥ P_90_. P_q_ = Percentile q.
